# Machine Learning in Modeling of Mouse Behavior

**DOI:** 10.3389/fnins.2021.700253

**Published:** 2021-09-14

**Authors:** Marjan Gharagozloo, Abdelaziz Amrani, Kevin Wittingstall, Andrew Hamilton-Wright, Denis Gris

**Affiliations:** ^1^Department of Neurology, Johns Hopkins University, Baltimore, MD, United States; ^2^Department of Pediatrics, Faculty of Medicine, Université de Sherbrooke, Sherbrooke, QC, Canada; ^3^Department of Radiology, Sherbrooke Molecular Imaging Center, Université de Sherbrooke, Sherbrooke, QC, Canada; ^4^School of Computer Science, University of Guelph, Guelph, ON, Canada; ^5^Department of Pharmacology and Physiology, Faculty of Medicine, Université de Sherbrooke, Sherbrooke, QC, Canada

**Keywords:** machine learning, behavior, home-cage ethome, computer modeling, circadian rythm

## Abstract

Mouse behavior is a primary outcome in evaluations of therapeutic efficacy. Exhaustive, continuous, multiparametric behavioral phenotyping is a valuable tool for understanding the pathophysiological status of mouse brain diseases. Automated home cage behavior analysis produces highly granulated data both in terms of number of features and sampling frequency. Previously, we demonstrated several ways to reduce feature dimensionality. In this study, we propose novel approaches for analyzing 33-Hz data generated by CleverSys software. We hypothesized that behavioral patterns within short time windows are reflective of physiological state, and that computer modeling of mouse behavioral routines can serve as a predictive tool in classification tasks. To remove bias due to researcher decisions, our data flow is indifferent to the quality, value, and importance of any given feature in isolation. To classify day and night behavior, as an example application, we developed a data preprocessing flow and utilized logistic regression (LG), support vector machines (SVM), random forest (RF), and one-dimensional convolutional neural networks paired with long short-term memory deep neural networks (1DConvBiLSTM). We determined that a 5-min video clip is sufficient to classify mouse behavior with high accuracy. LG, SVM, and RF performed similarly, predicting mouse behavior with 85% accuracy, and combining the three algorithms in an ensemble procedure increased accuracy to 90%. The best performance was achieved by combining the 1DConv and BiLSTM algorithms yielding 96% accuracy. Our findings demonstrate that computer modeling of the home-cage ethome can clearly define mouse physiological state. Furthermore, we showed that continuous behavioral data can be analyzed using approaches similar to natural language processing. These data provide proof of concept for future research in diagnostics of complex pathophysiological changes that are accompanied by changes in behavioral profile.

## Introduction

In neuroscience, understanding and evaluating mouse behavior are fundamental to the study of brain functions. Furthermore, mouse behavioral outcomes provide crucial evidence regarding the efficacy of new therapies (Jerndal et al., 2010; Richardson, 2015; Vuka et al., 2018). Most research designs include one or more behavioral tasks that are tailored to test specific functions (Bogdanova et al., 2013; Gregory et al., 2013). These tests, which usually last 5–15 min, require several independent observers who are blind to experimental conditions (Hanell and Marklund, 2014). Consequently, such tests are inherently sensitive to acclimating procedures and the expertise of the observers. To overcome the latter issue, testing procedures are recorded so that behavioral analysis can be performed after the fact and repeated several times. To address the need for data analysis, many free and commercially available tracking software packages have been developed (Richardson, 2015; Gris et al., 2017). Software solutions for automated behavioral assessment must address two inherent issues. First, automated assessment produces data at high frequency; most software packages require 30 frame-per-second resolution, yielding close to 3,000,000 data points per day. Second, automation provides the opportunity to analyze multiple behavioral activities, with some commercially available algorithms capable of characterizing more than 40 distinct mouse behavioral activities (Gris et al., 2017).

Although machine learning (ML) has been adopted in the field of biomedical research, it is not used in the analysis of animal behavior outcomes except in the detection of behavioral components and activities, so-called behavior segmentation. Comparatively little effort has been devoted to developing methods for analyzing numerical output data as a whole. Human intelligence is incapable of comprehending the distribution of variables in such a multi-dimensional data space. Accordingly, as researchers, we tend to reduce dimensionality and generalize data, narrowing down to the features that differ the most between control and experimental conditions. To help deal with the increasing complexity of the data space, several dimensionality-reduction approaches have been applied. Techniques such as principal component analysis (PCA), factor analysis, and various clustering algorithms can reduce 40-dimensional data down to four or five features (Gris et al., 2018; Gupta et al., 2018). Subsequently, these groups can be labeled with names that make sense in terms of the experimental setup (Richardson, 2015). For example, in daily mouse routine, we define groups of sleep-associated activities, physical activities, feeding activities, etc. (Yamamoto et al., 2018). These types of analyses have greatly facilitated consideration of a data set as a whole without requiring the analyst to select the most prominent features (Gris et al., 2018; Yamamoto et al., 2018). However, for this technique to be used, the data must be summarized by relatively large (hours/days/weeks) time windows, and different window sizes will contain different groups of behavioral activities. For example, a clustering algorithm will run differently on data summarized by days vs. hours, and in any case, the sequential nature of behaviors will be lost. Consequently, behavioral data pose an extremely complex problem for analysis. The best-known definitions of animal behavior that incorporate spatiotemporal characteristics are sexual courtship and bird song (Fee et al., 2004; Anholt et al., 2020). Analysis of these behaviors was originally performed by hand, requiring years of study in multiple laboratories.

Here, we describe several ML approaches that can be used to address the complexity of highly granulated time series data while maintaining the number of analyzed features. We examined three well-known, state-of-the-art approaches: logistic regression (LG), random forest (RF), and support vector machines (SVM). Although these algorithms take into account the behavioral content of a given time window, they do not consider sequential nature of the data. Therefore, we adopted recently discovered one-dimensional convolution networks and long short-term memory networks (1DConvLSTM) (Dauphin et al., 2017) algorithm used in natural language processing (NLP), in which the order of letters (sequence of events) within words, words within sentences, are crucial for analysis (Hirschberg and Manning, 2015). We hypothesized that due to the sequential understanding of input data embodied in the algorithm, deep learning algorithms consisting of 1DConvLSTM could accurately classify animal behavior. To test this approach, we sought to find the optimal way to classify mouse behavior into day and night sub-ethomes; an ethome describes the full set of observed behaviors of a single animal (Gris et al., 2017). This work provides proof-of-concept models for determination of day vs. night from behavior; inferences with final temporal resolution will be determined based on further study.

## Materials and Methods

Using mouse behavioral data collected at the University of Sherbrooke, we evaluated several ML algorithms for their ability to predict daytime versus nighttime behavior in mice. We evaluated a selection of algorithms individually and established a minimum window size for optimal characterization of day/night behavior.

All protocols and procedures were approved by the University of Sherbrooke Animal Ethics Use Committee protocol number 354-18. We used 8-week-old female C57Bl6 mice purchased from Jackson laboratories. Mice were recorded using a Swan surveillance camera system (with a 30 fps acquisition rate), and the data were analyzed using the CleverSys software as previously described (Gris et al., 2018; Yamamoto et al., 2018). The CleverSys software uses a computer-supervised method based on a hidden Markov model to analyze continuous video recordings and assign behavioral activities to sequences of mouse movements. Our previous findings suggested that in 10-day recordings, behavioral data between days 2 and 9 were most consistent (Gris et al., 2018; Yamamoto et al., 2018). We used this period in two identical experiment/video recordings performed several months apart with six mice per group; thus, a total of 12 mice were recorded. Previous studies confirmed the accuracy of behavioral data labeling by CleverSys algorithms (Gris et al., 2018; Yamamoto et al., 2018). The ground truth of day/night labeling was established based on a 10-h day/14-h night regime in the animal facility. The machine-learning problem was to predict day/night state based on a window of sequential samples drawn from the CleverSys labeled data.

The frames of the original video were much higher than required (33.3 Hz), so we reduced the effective frame rate in the CleverSys data. To this end, we created samples of mouse behavioral data by collecting sequences of 100 consecutive original frames, with a 3-s period for each data sample. For each sample, a label was established by applying the longest-duration CleverSys behavioral label over the entire sample.

Overlapping windows were then created to evaluate whether a 5-min (300-s) interval could provide sufficient information to establish a day/night labeling. Overlapping windows were created starting at each sample for a total of ∼26,000 time steps of 3 s (rows) with 37 behavioral activities per time point (columns), associated with an overall window label of “day” or “night”. In this manuscript, we report results for this 5-min window; earlier experiments evaluated a series of potential time periods.

Using this data as input, we evaluated accuracy of “day/night” prediction by four individual ML algorithms: LG, RF, SVM, and 1DConvBiLSTM. LG is one of the most popular statistical models for binary classification of multivariate data sets using probabilistic approach. RF is a classification and regression method based on a split decision at every tree that returns the classification selected by the most trees. SVM is another classification algorithm that recently gained popularly as it divides the data based on the largest separation margin.

Of these four classifiers, three (LG, RF, and SVM) constitute the battery of the primary methods of classification in biomedical research (Raevsky et al., 2018). They treat data as a tabular input vector, with no internal representation of a sample-to-sample sequential structure across the vector. In 1DConvBiLSTM, on the other hand, this sample-to-sample sequential representation is inherent in the algorithm itself; consequently, it can only be used on sequential data. Hence, we evaluated the ability of an ensemble classifier composed of all three of the tabular input algorithms to determine whether allowing the three algorithms to work together would balance the weaknesses of one against the other. Because our ensemble contains an odd number of classifiers (3) no tie-breaking strategy is needed for two-class labeling.

The code was implemented using the following open-source Python libraries: *os, sys*, and *re* (Rosenberg and Horn, 2016) for handling files and directories; *numpy* and *pandas* for data preprocessing*; keras* (Sato et al., 2018), and *tensor flow* (Rampasek and Goldenberg, 2016) for 1DConvBiLSTM*; scikit learn* (Abraham et al., 2014) for LG, SVM, and RF*;* and *mlstexnd* for ensemble methods. Graphs were built in R using the *dplyr* and *ggplot2* libraries (Ito and Murphy, 2013; Hicks and Irizarry, 2018). Model performance was evaluated by 5-fold cross-validation. In κ-fold cross-validation, model performance is evaluated by randomly dividing the training set into κ sets. One of these sets is left for testing, whereas the remaining κ-1 sets are used for training. The procedure is repeated κ times (Couronne et al., 2018). This method is often used to tune hyperparameters of ML algorithms with κ between 5 and 10.

## Results

### Day and Night Behaviors Differ in Wild-Type Mice

**FIGURE 1:**
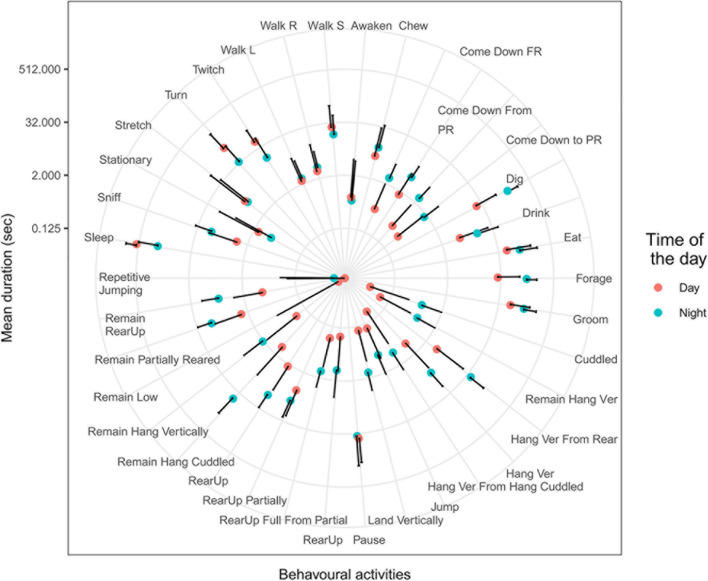
Behavioral activities during day and night. Mean and SD of behavioral activities during 5-min windows during day (red dots) and night (blue dots). Durations of more than 20 activities were statistically different between day and night (*T*-test, *p* < 0.001). Definition of behavioral activities described in Supplementary Table 1.

As an initial experiment, we summarized durations of various mouse behavior reported by CleverSys within windows of 5 min and 30-s intervals of activity and ran summary statistics comparing day and night. Durations of more than 25 behavioral activities differed between day and night (Figure 1). These data suggest that time intervals of 30 s could be informative for classifying behavior as day or night. However, not all mouse behavioral activities occur within windows of 30 s. For reference, the longest behavioral activity was “*Sleep*”, lasting 460 s, and the shortest were “*Dig*” and “*Awaken*”, both lasting 0.03 s. Although mice sleep more than 300 s only during the daytime, they do so no more than two or three times per day. The rest of the time, mice sleep for shorter intervals (Figure 1), making duration of a sleep alone a poor predictor of time of the day. Furthermore, the mean number of activities within 5-min windows was similar (*p* = 1) between day (225 ± 64) and night (263 ± 84), suggesting that the mouse behavioral repertoire does not significantly change over 24 h. Based on this observation, we used a 5-min window for further study.

We hypothesized that not only the duration of activities but also the combination of certain activities within a given time window would improve prediction accuracy. To explore this possibility, we chose to summarize 100 frames in 3-s summaries. Consequently, each day of the recording was represented by ∼26,000 time steps of 3 s (rows) with 37 behavioral activities per time point (columns). To determine whether the behavioral content in 5-min windows would be sufficient to accurately predict day vs. night, we oriented the representation such that each row contained data from 100 time steps (3 s per step × 100 steps = 300 s, i.e., 5 min). The resultant matrix consisted of 3,700 columns and ∼26,000 rows per mouse per day. We then used these data to build classification models of day and night using LG, RF, and SVM. To fine-tune hyperparameters in each algorithm, we performed a grid search with 5-fold cross validation within the training set.

### Logistic Regression

Logistic regression was optimized using 20% of the data, and the following parameters were selected using a grid search approach: *C* = 0.1, newton-cg solver function, and L2 regularization. We used L2 regularization because data have many colinear features. The optimized model was trained on 50% of the data, and the remaining 30% was used for testing.

**FIGURE 2:**
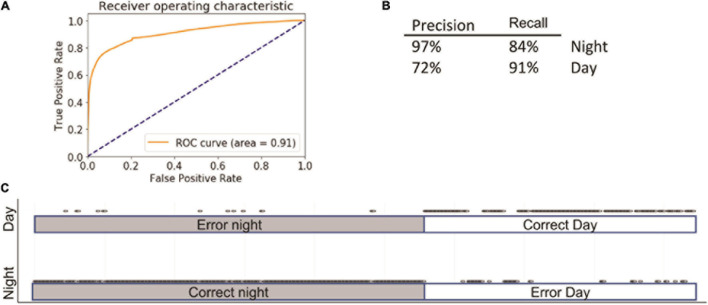
Summary of logistic regression (LG). Inserts show receiver operating characteristic (ROC) **(A)** curve, precision summary **(B)**, and graphical representation of the ratio of true positives to false positives **(C)**. Open circles represent predictions. The majority of predictions are correct; consequently, the circles overlap into apparent lines. Dots above marked bars represent incorrect predictions.

Using these parameters, we were able to predict day or night with 86 ± 1% accuracy; the area under the receiver operating characteristic (ROC) curve (AUC) was 0.91 (Figure 2A). The specificity and sensitivity of the model were 97% and 84% at night and 72% and 91% for the day (Figure 2B). The largest number of mistakes involved misclassifying day as night. The ROC curves of training and test did not differ (Figure 2C), indicating that the model was not overfitting the training data.

**FIGURE 3:**
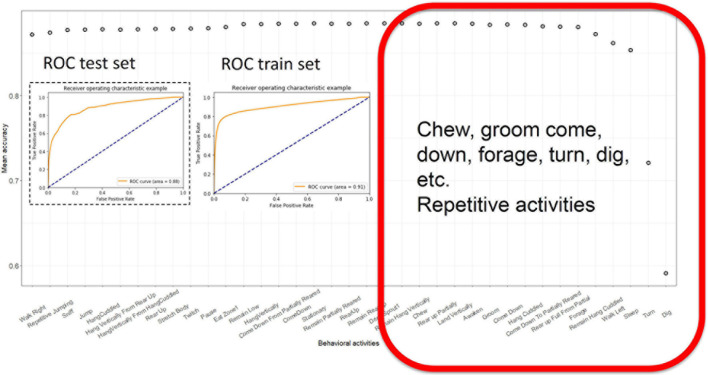
Backward feature elimination using LG. Each prediction model was computed, dropping the most irrelevant or confounding feature and least successful model was eliminated. Ultimately, when a few features remained, the models performance started to decline. The ROC curves of test and training sets with most successful model with least number of features. The red square contains a list of features that contribute the most to accuracy of the model.

We then used a backward feature elimination algorithm to select the most informative features. We evaluated the contribution of each feature to the accuracy of the model. Initially, we used 36 features and ran the LG model 36 times, omitting each feature individually. The feature with the worst contribution was discarded, and the model was evaluated with the 35 remaining features. This process was iterated until the performance of the LG classifier started to decline (Figure 3). At that point, the features remaining were “*Sleep*,” “*Turn*,” “*remain.Hang.Cuddled*,” “*Walk.Left*,” *“Dig*,” and “*Forage.”* These five features alone yielded 85 ± 1% accuracy (Figure 3).

### Random Forest

**FIGURE 4:**
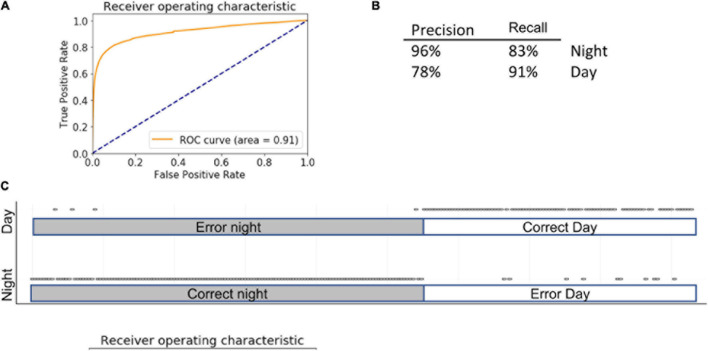
Summary of RF. Inserts show ROC **(A)** curve, precision summary **(B)**, and graphical representation of the ratio of true positives to false positives **(C)**. Open circles represent predictions. The majority of predictions are correct, and therefore circles overlap into apparent lines. Dots above marked bars represent incorrect predictions.

The RF method is based on the generation of multiple decision trees with random feature selection. RF has gained popularity due to its predictive power and is often considered as a standard method in classification tasks (Couronne et al., 2018). Grid search RF optimization yielded a model with following parameters: n_estimators = 80, max_features = “sqrt,” min_samples_leaf = 40, n_jobs = −1. Introducing various ccp_alpha values did not affect algorithm performance (data not shown). With cross validation cv = 5, the accuracy of the model was 87 ± 2%, AUC = 0.91 (Figure 4). Because it is impractical to use backward feature elimination in RF, we used the features that were selected in LG; accuracy remained similar (86 ± 9%) (Figure 4). There are two feature-importance functions within the RF algorithm. The first, based on the decrease in impurity at each tree, is calculated based on a training set. The second, based on feature permutation, can be implemented on both training and test sets and is therefore, more informative. In both cases, algorithms converged on features similar to those identified by LG, including *dig, forage*, and *sleep.* In addition, feature permutation selected for *eat, stretch*, and *hang cuddled.*

### Support Vector Machine

**FIGURE 5:**
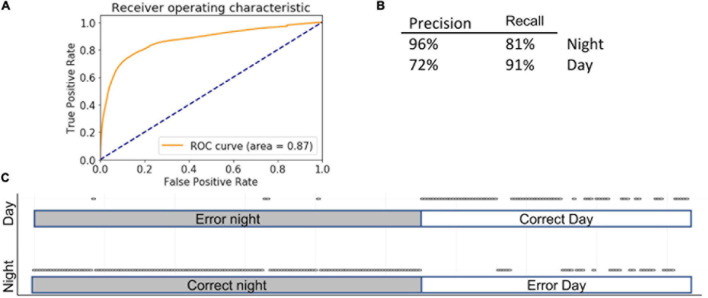
Summary of SVM. Inserts show ROC **(A)** curve, precision summary **(B)**, and graphical representation of the ratio of true positives to false positives **(C)**. Open circles represent predictions. The majority of predictions are correct, and therefore circles overlap into apparent lines. Dots above marked bars represent incorrect predictions.

While LG uses linear function to calculate separate probabilities (in case of categorical data), SVMs use geometrical properties of the data, maximizing the margin zone that separates classes. SVMs are computer-supervised methods for classifications and regressions that compute hyperplanes that separate the data in several ways based on the shape of the decision function. SVMs are inherently less sensitive to outliers than LG and perform better than RF on sparse data. In our case, encoding various behavioral activities over 5-min time windows yielded 3,600 columns. Therefore, an SVM design is better suited for such a problem. Fine-tuning hyper parameters yielded the following settings: kernel = “rbf” (recommended for high-dimensional data), gamma = 1, *C* = 10, decision_function_shape = “ovo.” Accuracy was 85 ± 5% using the full complement of features (AUC = 0.87) and 87 ± 2% using selected features (Figure 5).

### Ensemble

**FIGURE 6:**
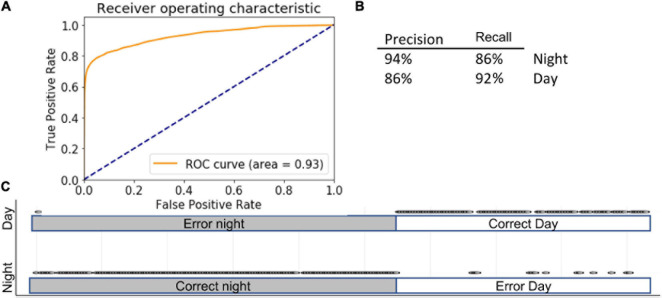
Ensemble. Inserts show ROC **(A)** curve, precision summary **(B)**, and graphical representation of the ratio of true positives to false positives **(C)**. Open circles represent predictions. The majority of predictions are correct, and therefore circles overlap into apparent lines. Dots above marked bars represent incorrect predictions.

We noted that each ML model made several mistakes that did not occur in other modes. Therefore, we employed a majority-vote ensemble method across the three different models, assuming that if two models were correct, the prediction would be more accurate. In ensemble methods, weight can be assigned to each model; in this case, however, because all models performed comparatively well, we allowed each an equal contribution of each model. Combining the models in this manner further improved the accuracy of distinguishing day from night (Figure 6): 90 ± 1% using the full complement of features (AUC = 0.93) and 90 ± 3% using selected features (Figure 6).

### 1DCBiLSTM

**FIGURE 7:**
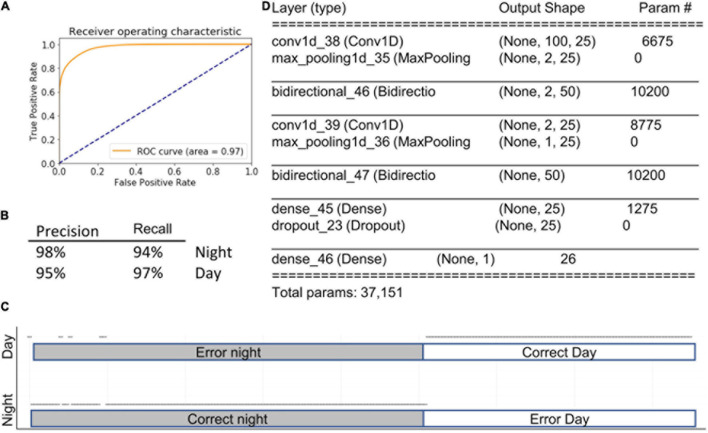
1DConvBiLSTM. Inserts show ROC curve **(A)**, accuracy metrics **(B)**, confusion matrix **(C)**, and summary of the model **(D)**. Open circles represent predictions. The majority of prediction are correct, and therefore circles overlap into apparent lines. Dots above marked bars represent incorrect predictions.

All of the outcomes from the algorithms described above are based on the presence of various behavioral activities within a selected time window. Importantly, the order in which features occur is not taken into account. Therefore, a sequence of imaginary features such as *a, b, c*. will yield the same result as *c, a, b*. Simple recurrent neural networks calculate the relationships among previous events in the most recent past. LSTM goes one step further, looking at both the distant and most recent past. Bidirectional LSTM runs the algorithms in two directions, from past and from future to the present. Therefore, we hypothesized that if the sequence in which features occurs is important for predicting physiological state, then bidirectional LSTM will outperform all other models. To reduce the complexity of the data we paired LSTM with 1DCONV networks. This strategy yielded a model with the highest accuracy in the study, 96% ± 1% (AUC = 0.97), outperforming even the ensemble method described above. Notably, when this approach was applied using features selected by LG, accuracy decreased to 88% (Figure 7), indicating that 1DConvBiLSTM can utilize data across a wider set of features than is available to the simple LG model.

## Discussion

In this study, we addressed some of the challenges in analysis of multidimensional continuous behavioral data. We found that 5 min of observed mouse behavior was sufficient to predict whether the time period was associated with day or night. Not surprisingly, new deep learning ML algorithms that account for the timing of behavioral activities yielded the best accuracy. Nevertheless, well described and computationally less involved algorithms such as LG, RF, and SVM also modeled mouse behavior with high accuracy. This is the first study to use exhaustive multidimensional behavioral high frequency time series data for computer modeling of mouse behavior. Note that in this work, we did not try to substitute existing tools used in circadian rhythm research. Day and night activities were chosen due to the obvious historical, biological, and experimental differences between the two classes.

Mining of multidimensional behavioral time-series data is not trivial. Because each feature is a discontinuous variable, moving average-based approaches are not suitable. For example, stock market prices may start high when trading opens and decline over the course of the day. In behavior, when a mouse is sleeping, it is not performing any of the 36 other activities that we examined; thus, the other activities do not decline, but are by definition absent. In addition, although we found that when we summarized over 5-min windows, the lengths of many behavioral activities differed significantly between day and night, none of the activities in isolation can be used for classification. For example, mice walk, eat, and groom during both day and night. Reinforcing this point, we found that the mouse behavioral repertoire was similar between day and night. Therefore, we hypothesized that combinations of activities would have a better predictive value than durations of individual activities alone. The availability of easily accessible open-source code for Python and R enabled several studies that applied complex statistical tools to biomedical research (Abraham et al., 2014; Gupta et al., 2018; Wang et al., 2019). For classification, the first approaches we applied to data were LG, RF, and SVM (Rashidi et al., 2019), which use different statistical approaches and thus complement each other. Note that we selected only three algorithms, the minimum required for a majority-vote ensemble procedure. Other methods that are often used in classification tasks include PCA, naïve Bayes, XG-boost, nearest mean, decision trees, and various clustering approaches.

Logistic regression is least flexible but the quickest and least computationally expensive method for modeling (Nick and Campbell, 2007). As the name suggests, this algorithm searches for linear relationships within the data. In our study, durations of more than 25 behavioral activities differed significantly between day and night, suggesting linear relationships within the data. In addition, LG allows for feature selection algorithms such as backward feature elimination. We used intervals of 100 time steps or 3 s, which increased the number of features to 3,700. Therefore, elimination had to occur before construction of the 5-min windows. This makes it challenging to use LG-embedded functions such as *lasso* or *elastic net*. In this study, most of the selected behavioral activities that contributed to the high accuracy of LG, aside from *sleep*, can be described as repetitive activities, and within this group, exploratory activities are the most prominent. Thus, this method provides the opportunity to select features that play the most significant role in the experimental setup. Notably, in our studies we did not account for two assumptions required for LG computation: only meaningful features should be included, and the variables should be independent of each other. Although meaningfulness was demonstrated in the backward feature elimination procedure, we know that behavioral variables were interrelated.

We continued to analyze data using other frequently used methods, including RF and SVM. SVM computes hyperplanes that separate classes (Heikamp and Bajorath, 2014), whereas RF is based on the generation of multiple decision trees with random feature selection (Sarica et al., 2017); accordingly, both can account for non-linear trends. In this study, RF and SVM performed similarly to LG. These three algorithms use different computational approaches and therefore are often used in an ensemble (Somasundaram and Alli, 2017) approach, as we did here. The ensemble method is based on the assumption that different models make mistakes at different times; therefore, combining several models together helps eliminate mistakes, provided that other models have made correct predictions. Because different models are based on different modes of data analysis, it is typical that the assumptions of these model differ. Using an *ensemble* method allows several ML algorithms to be aggregated together to overcome the weakness of a single one, with the weakest learning assessed separately for each data point. Put another way, one of the models may have learned a particular aspect of the problem well, but when the data do not highlight that aspect of the problem, it performs more poorly. When placed in an ensemble, its contribution varies based on whether other models agree on each point, and different pairings of models may be correct for two different data points.

The scikit library enables us to compare the results of all three algorithms separately and in combination (Abraham et al., 2014), making it easy to explore the contribution of this mixed model. Although all three algorithms, LG, SVM, and RF, yielded excellent results on their own, the ensemble method achieved a modest improvement. AUC values of test and training sets remained close for all models, indicating that algorithms did not overfit the data.

Next, we wanted to incorporate the temporal pattern into our analysis of mouse behavior. LG, SVM, and RF analyzed the combination of behavioral activities in 5-min windows without taking into consideration the sequence of those activities. Behavioral data are reminiscent of language, which is also exhaustive and self-exclusive: exhaustive because a word must contain letters derived from the alphabet for that language, and self-excusive because if one letter appears at a given moment, another letter cannot appear at the same time. Although the word is a continuous event, each letter is a discrete event, and collectively these discrete events form patterns. These types of data are not amenable to classical time-series data mining algorithms based on moving averages. Therefore, to dissect behavioral temporal patterns, we applied insights from the NLP field. Over years of study in multiple laboratories, temporal movement patterns were studied and used to define complex activities such as grooming (Kalueff et al., 2016). Thus, mouse grooming was defined as a sequence of movement starting with licking the paws, cleaning the nose and nose area with both paws, cleaning whiskers with a single paw, and brushing the head with two paws; later, the mouse cleans the torso, and finally the urogenital area and tail. Functionally, grooming is associated with thermoregulation, wound healing, cleaning, self-soothing under stress, and elimination of louse colonies (Spruijt et al., 1992; Kalueff and Tuohimaa, 2004; Kalueff et al., 2007). Although grooming is just one of many measured features in our study, the microstructure of grooming has become a research field in itself (Kalueff et al., 2007).

It is challenging to analyze large sets of time-series behavioral data. A study by Wiltschko et al. (2015) used Bayesian non-parametric and Markov Chain Monte Carlo (MCMC) models to construct and predict short mouse behavioral modules and transitions. Using 3D tracking of 20-min videos taken in the open field, the authors adopted an unsupervised computational approach to identify new behavioral structures and mouse postures at sub-second levels; in addition, they were able to construct short sequences of mouse behavioral transitions. The main contribution of that study was the use of novel image processing/analysis in defining behavioral activities, with digital imaging as variables (Wiltschko et al., 2015). By contrast, in this study we capitalized on algorithms that define behavioral content consisting of sequences of defined behavioral activities. The main distinguishing characteristics of our approach are that we used prior knowledge in defining behavioral structures (computer-supervised method) over much longer time frames, and used multiple behavioral activities as variables. Our work suggests that the sequence of these behavioral modules (in our case, human-defined) forms patterns that constitute distinct behavioral routines on a 1,440-min scale in a home-cage setting.

We hypothesized that mouse behavior during the day and night differs in duration, frequency, and order/sequence of behavioral components and activities. We used recurrent neural networks to associate the time-series data of 37 behavioral activities to define time of day. Until the distribution of the tensor flow library, deep learning approaches were feasible only for a selected group of computer scientists with expertise in computation, statistics, and programming. With an increase in the abundance of open-source resources, deep learning libraries have become available even for amateur programmers from other scientific fields, including biomedical research (Nam et al., 2019). Although we can trace back the distribution of weights, the modeling of relationships between features remains a black box. Also, the back-elimination feature selection techniques used with LG is not practical due to the enormous computation times required and the disruption of the sequential order of activities excluded from the analysis. In our study, we combined 1DConv neural networks with BiLSTM recurrent deep neural networks; both of these algorithms are used in NLP (Li et al., 2018; Kilicoglu et al., 2019). Combining them into a single model is a relatively new idea. 2DConv networks have been used extensively in image processing due to its superb feature extraction ability. Later, 1DConv networks were introduced to NLP, which helped to improve the accuracy of the models. At the same time, recurrent networks were used across text to maintain chronological order. Both of these algorithms yielded good accuracies, but the introduction of hybrid algorithms that combined 1DConvBiLSTM improved accuracy even further (Kilicoglu et al., 2019; Li et al., 2019). 1DConv extracts the meaning of the words, whereas BiLSTM extracts the meaning of the sentences and paragraphs. In our opinion, NLP and behavioral analysis are similar: in the data set analyzed in this study, each activity can be considered as a letter; 1DConv detects patterns of different combinations of activities, whereas BiLSTM keeps track of chronological patterns and places them in “sentences” and “paragraphs” that define day- and night-specific behavior.

Unlike NLP, behavior has a “tempo” dimension to it, meaning that we can read texts at any speed we want or even pause for a day or so. In our opinion, behavior resembles music, in which tempo is crucially important. Although BILSTM can detect bidirectional temporal functions, we do not know what those patterns are. Further detailed analysis will be required to dissect the spatio-temporal nature of day and might mouse behavior.

## Conclusion

In this paper, we did not aim to describe all ML techniques known today. Rather, we demonstrated how several widely accepted approaches perform in exploring behavioral data space. We considered continuous behavioral data as time-series data, allowing us to compute precise predictive models. We also demonstrated that a simple feature selection tool that uses a drop-one-out method for backward feature elimination can drastically decrease the dimensionality of the data. This method provides a visual representation of how each feature contributes to the overall computational model, and thus helps to form hypotheses about biological mechanisms that underlie the experimental design. Dimensionality reduction was not among the goals of this paper. For example, the *sleep* activity may appear shorter since it alternates with *twitch*. The multidimensional nature of behavioral data decreases the importance of activity considered in isolation, thereby reducing bias and improving reproducibility. Of note, that our experimental set up didn’t used the long habituation periods. Only the first day of recording was discarded. Finally, our studies suggest similarity a between NLP and behavior analysis. Our previous studies showed that even small deviations in inflammatory status result in significant behavioral changes. The data flow and computational parameters established here will be useful for future descriptions of the mouse brain and systemic inflammation sub-ethomes.

## Data Availability Statement

The original contributions presented in the study are included in the article/Supplementary Material, further inquiries can be directed to the corresponding author.

## Ethics Statement

The animal study was reviewed and approved by Université de Sherbrooke Animal Ethics Use Committee.

## Author Contributions

MG, AA, KW, AH-W, and DG conceived the study. MG built the experiment with supervision from DG. DG and AH-W collected and analyzed the data. DG wrote the manuscript with input from MG, AA, and AH-W. All authors contributed to the article and approved the submitted version.

## Conflict of Interest

The authors declare that the research was conducted in the absence of any commercial or financial relationships that could be construed as a potential conflict of interest.

## Publisher’s Note

All claims expressed in this article are solely those of the authors and do not necessarily represent those of their affiliated organizations, or those of the publisher, the editors and the reviewers. Any product that may be evaluated in this article, or claim that may be made by its manufacturer, is not guaranteed or endorsed by the publisher.
