# Supplementary material for: Machine Learning in Modeling of Mouse Behavior
Source: Front Neurosci. 2021 Sep 14;15:700253. doi: 10.3389/fnins.2021.700253 (PMC8477014; doi:10.3389/fnins.2021.700253)
Supplement: Supplementary file 1 [file Table_1.PDF]

Supplemental Table 1. Definition of detected behavioral activities.

Activity	Definition	Abbreviation
Awaken	Any movement from sleep that starts and continues without resumption of sleep	Awaken
Chew	Any brief period during eating where the mouth detaches from the food	Chew
Come down	Any movement of the animal from a fully reared position to a position	Come Down
Come down from partially reared	Any movement of the animal from a partially reared up position to a low level	Come Down from PR
Come down to partially reared	Any movement of the animal from a fully reared up position to a partially reared up position	Come Down to PR
Dig	Any movement with animal's hind limbs inside bedding and resulting in a considerable movement of the bedding	Dig
Drink	In a reared up position. animal's nose/mouth crosses water calibrated line	Drink
Eat	In a reared up position, animal's nose/mouth crosses feeding box calibrated line	Eat
Forage	Any movement with animal's forepaws and/or mouth inside bedding and resulting in a considerable movement of the bedding, typically in front of the animal	Forage
Groom	Deformation of body over a defined criteria and longer than specified time	Groom
Hang cuddled	Any movement of the animal resulting in animal having all four limbs at the top of the cage (more horizontal position at the top of the cage)	Hang Cuddled
Hang vertically from hang cuddled	Any movement from a hang cuddled position to a hang vertical position	Hang Ver from Hanged Cuddled
Hang vertically from rear up	Any movement of the animal resulting in animal leaving the floor and not coming back<br>down immediately and remaining vertical after leaving the floor	Hang Ver From RearUp
Jump	Any movement from a lower to a higher position and back to the position	Jump
Land vertically	Any movement of the animal from a hanging position with feet off of the floor to the feet coming back down on to the floor	Land Ver
Pause	Implemented similar to sleep with those sleep constraints, but lasting only for much smaller prescribed minimum time	Pause
Rear up	Any movement of the animal from a low position to a full reared up position	RearUp
Rear up from partially reared	Any movement of the animal from a partially reared position to a fully reared up position	ReaurUp from PR
Rear up partially	Any movement of the animal from position to a partially reared position	RearUp Part
Remain hang cuddled	After hanging cuddled, remain in a hanging cuddled position	Remain Hang Cuddled
Remain hang vertically	After hanging vertically remain in a vertical position	Remain Hang Ver
Remain partially reared	Remain in a partially reared position	Remain PR
Remain reared up	After rearing up, remain in the reared up position	Remain RearUp
Repetitive jumping	Any series of successive jump behaviors	Repetitive jump
Sleep	The onset of sleep is detected at each instance there is no significant movement for a prescribed amount of time	Sleep
Sniff	When the animal is either fully or partially reared, and the tip of the mouth makes some random movements (back and forth, up/down, protrude/retract)	Sniff
Stationary	Any sequence for which there is no translational movement	Stationary
Stretch body	Any movement from shorter to longer/elongated body (horizontally or vertically)	Stretch
Turn	Any movement of the animal from a side view to a front vice versa	Turn
Twitch	Any brief movement of the animal during sleep	Twitch
Walk left	Any movement of the animal in left direction over a given distance	Walk L
Walk right	Any movement of the animal in right direction over a given distance	Walk R
Walk slowly	Any sideways movement of animal without a definite direction component	Walk S
