# Supplementary material for: Machine Learning in Modeling of Mouse Behavior
Source: Front Neurosci. 2021 Sep 14;15:700253. doi: 10.3389/fnins.2021.700253 (PMC8477014; doi:10.3389/fnins.2021.700253)
Supplement: Supplementary file 2 [file Data_Sheet_1.pdf]

Supplemental Data: Computer code

```
# This code summarizes several prediction algorithms including Logistic regression
#Random forest, Support vector machines, and ensemble
# For simplicity all libraries will be imported in the beginning of the code, not at the point of the first
#use.
import pandas as pd
import glob, os
import numpy as np
import time
import sys
from sklearn.model_selection import GridSearchCV
from sklearn.preprocessing import MinMaxScaler
from sklearn.preprocessing import LabelEncoder
from sklearn.linear_model import LogisticRegression
from sklearn import svm
from sklearn.ensemble import RandomForestClassifier
from mlxtend.classifier import EnsembleVoteClassifier
from sklearn.model_selection import cross_val_score

# To keep track of time that it takes to execute the code
Start = time.time()



def split_train_test(df, a, b, split, window):# df is data, a and b are
#strings like "day" and "night", split is a fraction between 0 and 1
#window deletes last rows in Y (dependent variable) to match X (independent variable) after dropna
# It reserves sequential manner of the data
#take a data frame with two times of the days .
#split them into train and test each and merges them back
#in the end it returns 2 sets train and test of day and night for X and Y
    df_a = df.loc[df["z"] == a]
    df_b = df.loc[df['z'] == b]
    win = int(2*window-1)
    a_l = len(df_a)
    a_l_s = int(len(df_a)*split)
    df_a_tr= df_a.iloc[0:a_l_s, :]
    df_a_te = df_a.iloc[a_l_s:a_l, :]
    X_a_train = df_a_tr.iloc[:, 0:-1]
    X_a_train = X_a_train.astype(np.float32)
    Y_a_train = df_a_tr.iloc[:-win, -1]
    X_a_test = df_a_te.iloc[:, 0:-1]
    X_a_test = X_a_test.astype(np.float32)
```

```
    Y_a_test = df_a_te.iloc[:-win, -1]
    b_l = len(df_b)
    b_l_s = int(len(df_b)*split)
    df_b_tr= df_b.iloc[0:b_l_s, :]
    df_b_te = df_b.iloc[b_l_s:b_l, :]
    X_b_train = df_b_tr.iloc[:, 0:-1]
    X_b_train = X_b_train.astype(np.float32)
    Y_b_train = df_b_tr.iloc[: -win, -1]
    X_b_test = df_b_te.iloc[:, 0:-1]
    X_b_test = X_b_test.astype(np.float32)
    Y_b_test = df_b_te.iloc[: -win, -1]
    l =[X_a_train, X_a_test, Y_a_train, Y_a_test,
     X_b_train, X_b_test, Y_b_train, Y_b_test]
    return(l)

#Slide window function defines number activities in the window. During data preprocessing step
#activities were summarized by 3 seconds, so window of a 100 defines 5 minutes.

def slide_window(df, window):

    holder = pd.DataFrame()
    nrows = df.shape[0]

    for i in range(0, window):
        a = df.iloc[i+window:nrows, :]
        a = a.reset_index(drop = True)
        holder = pd.concat([holder, a], axis = 1, ignore_index = True)

    holder = holder.dropna()

    return holder
#load data frame
file = ‘file_name’
file1 = 'female_2_3_4_2017.csv'
#read file
df1 = pd.read_csv(file1)
#show list of activities
column = list(df1.columns)
print(column)
df2 = df2[['Sleep', 'Twitch', 'Turn', 'Awaken', 'Groom', 'Stationary', 'Come.Down.From.Partially.Reared',
'Rear.up.Partially', 'Remain.Partially.Reared', 'Stretch.Body', 'Sniff', 'Eat.Zone1', 'Walk.Right',
'Walk.Slowly', 'Drink.Spout1', 'Walk.Left', 'Rear.up.Full.From.Partial', 'Come.Down', 'Remain.RearUp',
'RearUp', 'Dig', 'Jump', 'Hang.Vertically.From.Rear.Up', 'Forage', 'Come.Down.To.Partially.Reared',
'HangVertically', 'Land.Vertically', 'ComeDown', 'HangCuddled', 'Chew', 'Rear.Up',
```

```
'Remain.Hang.Cuddled', 'Pause', 'HangVertically.From.HangCuddled', 'Remain.Hang.Vertically',
'Hang.Cuddled', 'Repetitive.Jumping', 'z', 'day']]
#Select data excuding last column
df = df.iloc[:, 0:-1]
#Define window size
window = 100

[X_a_train, X_a_test, Y_a_train, Y_a_test,
X_b_train, X_b_test, Y_b_train, Y_b_test]  = split_train_test(df, "day", "night", 0.5 , window)
l_df = [X_a_train, X_a_test, X_b_train, X_b_test]
l_df_t = list()
#add and concatenate window
X_day_train = slide_window(X_a_train, window)
X_day_test = slide_window(X_a_test, window)
X_night_train = slide_window(X_b_train, window)
X_night_test = slide_window(X_b_test, window)


#define variables if window is used
X = pd.concat([X_day_train, X_night_train], axis = 0, ignore_index = True)
X_test = pd.concat([X_day_test, X_night_test], axis = 0, ignore_index = True)
y = pd.concat([Y_a_train, Y_b_train], axis = 0, ignore_index = True)
Y_test= pd.concat([Y_a_test, Y_b_test], axis = 0, ignore_index = True)

#Scaling the data
scaler = MinMaxScaler(feature_range = (0,1))
scaler.fit(X)
X= scaler.transform(X)
X_test = scaler.transform(X_test)

# Encoding y day and night
labelencoder_y = LabelEncoder()

Y_a_train = labelencoder_y.fit_transform(Y_a_train)
Y_b_train = labelencoder_y.fit_transform(Y_b_train)
Y_a_test = labelencoder_y.fit_transform(Y_a_test)
Y_b_test = labelencoder_y.fit_transform(Y_b_test)
y = labelencoder_y.fit_transform(y)
Y_test = labelencoder_y.fit_transform(Y_test)

## building a simple linear regression model fine tuning example using GridSearch library
solver = ['newton-cg', 'sag','saga', 'lbfgs', 'liblinear']
C = [0.01, 0.1, 1, 10, 100, 1000]
penalty= ['l1', 'l2']
```

```
#

# param_grid = dict(penalty = penalty, solver =  solver)
# grid = GridSearchCV(estimator = grid_search_l, param_grid = param_grid, n_jobs=1, cv=5)
# grid_result = grid.fit(X,y)

# print("best: %f using %s" % (grid_result.best_score_, grid_result.best_params_))
# means = grid_result.cv_results_['mean_test_score']
# stds = grid_result.cv_results_['std_test_score']
# params = grid_result.cv_results_['params']
# for mean, stdev, param in zip(means, stds, params):
#    print('%f (%f with %r' % (mean, stdev, param))

# # Using already tuned logistic regression classifier
classifier_l = LogisticRegression( random_state = 0, solver = "newton-cg", C = 0.01, penalty = 'l2')


#Buildind SVM
#gamma can be between 0.1 and a 100 larger number leads to more computing time
#kernel can be set to kernel = ['rbf', 'poly','sigmoid']
# with 'rbf' C = [0.1, 1, 10, 100, 1000]
#with 'ploly' degrees = [1, 2, 3, 4, 5, 6, 7]


 grid_search_s = svm.SVC()

#Tuning the model
kernel= ['rbf', 'poly','sigmoid']
decision_function = ['ovr', 'ovo']
param_grid = dict(kernel = kernel)
grid = GridSearchCV(estimator = grid_search_s, param_grid = param_grid, n_jobs=1, cv=5)
grid_result = grid.fit(X,y)

print("best: %f using %s" % (grid_result.best_score_, grid_result.best_params_))
means = grid_result.cv_results_['mean_test_score']
stds = grid_result.cv_results_['std_test_score']
params = grid_result.cv_results_['params']
for mean, stdev, param in zip(means, stds, params):
print('%f (%f with %r' % (mean, stdev, param))

#Model after finetuning
classifier_s = svm.SVC(kernel = 'rbf',gamma = 1, C = 10, decision_function_shape = 'ovo')
```

```
#Building Random Forest
#number of tree n = [5, 20, 40, 80, 120, 200]
#maximal_features = [0.1, 0.4, 0.8] is a proportion of all features at one tree
# minimal_sample_leaf around 50 , need to try several inceases noise
from sklearn.ensemble import RandomForestClassifier
classifier_r = RandomForestClassifier(n_estimators = 100)
#grid = GridSearchCV(estimator = neural_network, param_grid = param_grid, n_jobs=1)
#grid_result = grid.fit(X,y)

#cross validation

from sklearn.model_selection import cross_val_score

cv = 5
scores = cross_val_score(classifier_s, X, y, cv=cv, scoring = "accuracy")
scores1 = cross_val_score(classifier_s, X, y, cv=cv, scoring = "precision_macro")
scores2 = cross_val_score(classifier_s, X, y, cv=cv, scoring = "recall_macro")
scores3 = cross_val_score(classifier_s, X, y, cv=cv, scoring = "f1_macro")

mean_accuracy = scores.mean()



#arranging results
results = pd.DataFrame(np.array([scores, scores1, scores2, scores3]))
results = results.T
results.columns = ("accuracy", "precision", "recall", "f")
results.to_csv('s_%s'%file3)

#Building majority vote classifier- ensemble aproach
eclf = EnsembleVoteClassifier(clfs=[classifier_l, classifier_s, classifier_r], weights=[1, 1, 1])
fit = eclf.fit(X, y)
y_pres = pd.DataFrame(fit.predict(X_test))
#y_pres.to_csv("y_famleodd_w_%d2_vote.csv"%window)

cv = 5
scores = cross_val_score(fit, X, y, cv=cv, scoring = "accuracy")
print(scores.mean())


result = pd.DataFrame()
labels = ['Logistic Regression', 'SVM', 'Random Forest', 'Ensemble']
```

```
for clf, label in zip([classifier_l, classifier_s, classifier_r, eclf], labels):
    scores = model_selection.cross_val_score(clf, X, y, cv=5, scoring='accuracy')
    scores = pd.DataFrame(scores)
    result = pd.concat([result, scores], axis = 1, ignore_index = True, sort = False)
    print("Accuracy: %0.2f (+/- %0.2f) [%s]" % (scores.mean(), scores.std(), label))
result.to_csv('result_%d2.csv'%window)
 # To keep track of time that it takes to execute the code
End = time.time()
Print(End – Start
```

This codes sumarises 1DConvBiLSTM

```
import pandas as pd

import numpy as np

import time


start = time.time()

df = pd.read_csv("name_of_the_file")

#Select data of behavioral activities

df = df[['Sleep', 'Twitch', 'Awaken', 'Groom', 'Stationary', 'Come.Down.From.Partially.Reared',
'Rear.up.Partially', 'Remain.Partially.Reared', 'Stretch.Body', 'Sniff', 'Eat.Zone1', 'Walk.Right',
'Walk.Slowly', 'Drink.Spout1', 'Rear.up.Full.From.Partial', 'Remain.RearUp', 'RearUp', 'Dig', 'Forage',
'Come.Down.To.Partially.Reared', 'HangVertically', 'Land.Vertically', 'ComeDown',  'Chew', 'Rear.Up',
'Remain.Hang.Cuddled', 'Pause', 'HangVertically.From.HangCuddled', 'Remain.Hang.Vertically',
'Hang.Cuddled', 'z']]


day = df.loc[df["z"] == 'day']

night = df.loc[df['z'] == 'night']

    #this split is for LSTM . It reserves sequential manner of the data

#take a data frame with two times of the days .

    #split them into train and test each


def split_train_test(df, a, b, split):
```

```
    df_a = df.loc[df["z"] == a]
    df_b = df.loc[df['z'] == b]
    a_l = len(df_a)
    a_l_s = int(len(df_a)*split)
    df_a_tr= df_a.iloc[0:a_l_s, :]
    df_a_te = df_a.iloc[a_l_s:a_l, :]
    X_a_train = df_a_tr.iloc[:, 0:-1]
    X_a_train = X_a_train.astype(np.float32)
    Y_a_train = df_a_tr.iloc[:, -1]
    X_a_test = df_a_te.iloc[:, 0:-1]
    X_a_test = X_a_test.astype(np.float32)
    Y_a_test = df_a_te.iloc[:, -1]
    b_l = len(df_b)
    b_l_s = int(len(df_b)*split)
    df_b_tr= df_b.iloc[0:b_l_s, :]
    df_b_te = df_b.iloc[b_l_s:b_l, :]
    X_b_train = df_b_tr.iloc[:, 0:-1]
    X_b_train = X_b_train.astype(np.float32)
    Y_b_train = df_b_tr.iloc[:, -1]
    X_b_test = df_b_te.iloc[:, 0:-1]
    X_b_test = X_b_test.astype(np.float32)
    Y_b_test = df_b_te.iloc[:, -1]
    return(X_a_train, X_a_test, Y_a_train, Y_a_test,
           X_b_train, X_b_test, Y_b_train, Y_b_test)

(X_dask_train, X_dask_test, Y_dask_train, Y_dask_test,
 X_dawn_train, X_dawn_test, Y_dawn_train, Y_dawn_test) = split_train_test(df, "day", "night", 0.5)
#building sliding window
window = 100
```

```
def slide_window(df_x, df_y,  window):
    z=window
    a=len(df_x)
    c = len(df_y)-z+1
    D=pd.DataFrame()
    for i in range(0, z):
        x = df_x.iloc[i:a, :]
        x = x.reset_index(drop = True)
        D = pd.concat([D, x], axis = 1, ignore_index = True)
        D = D.dropna()
    y = df_y.iloc[0:c]
    return(D, y)

(X_dask_train_1, Y_dask_train_1) = slide_window(X_dask_train, Y_dask_train, window )
(X_dask_test_1, Y_dask_test_1) = slide_window(X_dask_test, Y_dask_test, window)
(X_dawn_train_1, Y_dawn_train_1) = slide_window(X_dawn_train, Y_dawn_train,window )
(X_dawn_test_1, Y_dawn_test_1) = slide_window(X_dawn_test, Y_dawn_test,window )

X1 = pd.concat([X_dask_train_1, X_dawn_train_1], axis = 0, ignore_index = True)
Y = pd.concat([Y_dask_train_1, Y_dawn_train_1], axis = 0, ignore_index = True)
X_test = pd.concat([X_dask_test_1, X_dawn_test_1], axis = 0, ignore_index = True)
Y_test = pd.concat([Y_dask_test_1, Y_dawn_test_1], axis = 0, ignore_index = True)

#Encode the data
from sklearn.preprocessing import LabelEncoder
labelencoder_y_2 = LabelEncoder()
y = labelencoder_y_2.fit_transform(Y)
Y_test = labelencoder_y_2.fit_transform(Y_test)
```

```
#For BiLSTM we have to  transform feature variables into 3D array

X = X1.to_numpy()
X=X.reshape(len(X), window, int(X1.shape[1]/window))
X_test = X_test.to_numpy()
X_test = X_test.reshape(len(X_test), window, int(X1.shape[1]/window))
# building a LSTMmodel
# Importing the Keras libraries and packages
import keras
from keras.models import Sequential
from keras.layers import LSTM
from keras.layers import Bidirectional
from keras.layers import TimeDistributed
from keras.layers import Dense
from keras.layers import Dropout
from keras import models
from keras import layers
from keras.wrappers.scikit_learn import KerasClassifier
from sklearn.model_selection import GridSearchCV
from keras.layers import LeakyReLU
time_step = window
max_pool = int(window/2)
def create_network():
   classifier = Sequential()
   #adding a 1D convolution layer

   classifier.add(layers.Conv1D(15, 14, activation ='relu', padding = 'same', input_shape = (time_step,
X.shape[2])))
```

```
    #classifier.add(LeakyReLU(alpha = 0.03))
    classifier.add(layers.MaxPooling1D(max_pool))
    #classifier.add(layers.Conv1D(16, 7, activation = 'relu', padding='same'))
    # Adding the recurent layer

    classifier.add(Bidirectional(LSTM(15, return_sequences = True, dropout = 0.7, recurrent_dropout =
0.7, input_shape = (max_pool, 25)))) #,
    #classifier.add(Bidirectional(LSTM(36, return_sequences = True, dropout = 0.5, recurrent_dropout =
0.1)))
    classifier.add(layers.Conv1D(15, 7, activation = 'relu', padding='same'))
    classifier.add(layers.MaxPooling1D(2))
    classifier.add(Bidirectional(LSTM(15)))

    classifier.add (Dense(25, activation = 'relu'))
    classifier.add(Dropout(0.7))
    #adding output layer
    classifier.add(Dense(1, activation = 'sigmoid'))

    # Compiling the network
    adam = keras.optimizers.Nadam(lr = 0.002, beta_1 = 0.7, beta_2 = 0.999, )
    classifier.compile(optimizer = 'adam', loss = 'binary_crossentropy', metrics = ['accuracy'])

    return(classifier)

#building LSTM
#neural_network = KerasClassifier(build_fn = create_network, verbose=0, shuffle = False)
neural_network = create_network()
history=neural_network.fit(X,y, epochs = 50, batch_size = 400, validation_split = 0.2)
```

```
print(neural_network.summary())


show = pd.DataFrame(history.history)
show.tail()



# Predicting the Test set results
#X_test = dask_dawn_test.iloc[:, 0:-1]
#X_train = dask_dawn_train.iloc[:, -1]


y_pred_c = neural_network.predict_classes(X_test)
y_train_c = neural_network.predict_classes(X)
y_pred = neural_network.predict_proba(X_test)
y_train = neural_network.predict_proba(X)


# Making the Confusion Matrix, report, printing summary and plot the model
from sklearn.metrics import confusion_matrix
from sklearn.metrics import classification_report
from sklearn.metrics import accuracy_score
from keras.utils.vis_utils import plot_model
cm = confusion_matrix( y_pred_c, Y_test)
rpt = classification_report(y_pred_c, Y_test)


ac = accuracy_score(y_pred_c, Y_test)
print(ac)


#print(neural_network.summary())
```

```
#plot_model(neural_network, to_file = 'model_plot.png', show_shapes=True, show_layers_names = True)

pred = pd.DataFrame(y_pred)

#pred.to_csv("conv_lstm_99_2.csv")


# Learning metrics of the models

from sklearn.metrics import roc_curve, auc


from sklearn.preprocessing import label_binarize

from sklearn.multiclass import OneVsRestClassifier

from scipy import interp

from sklearn.metrics import roc_auc_score

from sklearn import metrics

fpr, tpr, thresholds = metrics.roc_curve(Y_test, y_pred)

roc_auc = auc(fpr, tpr)

## Compute micro-average ROC curve and ROC area

#fpr["micro"], tpr["micro"]= roc_curve(Y_test.ravel(), Y_pred.ravel())

#roc_auc["micro"] = auc(fpr["micro"], tpr["micro"])


from sklearn.model_selection import cross_val_score

cv = 5

#scores = cross_val_score(fit, X_test, Y_test, cv=cv, scoring = "accuracy")

#print(scores.mean())


import matplotlib.pyplot as plt

plt.figure()

lw = 2

plt.plot(fpr, tpr, color='darkorange',

        lw=lw, label='ROC curve (area = %0.2f)' % roc_auc)
```

```
plt.plot([0, 1], [0, 1], color='navy', lw=lw, linestyle='--')
plt.xlim([0.0, 1.0])
plt.ylim([0.0, 1.05])
plt.xlabel('False Positive Rate')
plt.ylabel('True Positive Rate')
plt.title('Receiver operating characteristic ')
plt.legend(loc="lower right")
plt.show()

#Looking at the loss function
#from keras.models import load_model
plt.figure(figsize=(8,4))
plt.plot(history.history['loss'], label='Train Loss')
plt.plot(history.history['val_loss'], label='Test Loss')
plt.plot(history.history['acc'], label = 'Accuracy')
plt.plot(history.history['val_acc'], label = 'Val_accuracy')
plt.title('model loss')
plt.ylabel('loss')
plt.xlabel('epochs')
plt.legend(loc='upper right')
plt.show();
#Saving and loading the model
neural_network.save( "%s.h5" %file_name)
# from keras.models import load_model
# neural_network1 = load_model( "file_name.h5")

end= time.time()
print(end-start)
```
